# Negative hyperselection of patients with stage III colon cancer receiving anti-EGFR-based adjuvant treatment

**DOI:** 10.1016/j.esmoop.2025.105857

**Published:** 2025-10-29

**Authors:** M. Ambrosini, H. Blons, S. Garinet, C. Mulot, D. Le Corre, S. Mouillet-Richard, F. Pietrantonio, M. Sroussi, C. Lepage, P. Laurent-Puig, J. Taieb

**Affiliations:** 1 Institut du Cancer Paris Carpem, Assistance Publique-Hôpitaux de Paris, Université Paris Cité, Department of Gastroenterology and Gastrointestinal Oncology, Georges-Pompidou European Hospital, Paris, France; 2 Centre de Recherche des Cordeliers, INSERM, CNRS, Université Paris Cité, Sorbonne Université, USPC, Equipe labellisée Ligue Nationale Contre le Cancer, Paris, France; 3 Department of Medical Oncology, Fondazione IRCCS Istituto Nazionale dei Tumori, Milan, Italy; 4 Assistance Publique-Hôpitaux de Paris, Department of Biochemistry, Pharmacogenetics and Molecular Oncology, European Georges-Pompidou Hospital, Paris Cancer Institute CARPEM, Paris, France; 5 Institut du Cancer Paris Carpem, Assistance Publique-Hôpitaux de Paris, Université Paris Cité, Genomic Medicine of Tumors and Cancers Department, Georges-Pompidou European Hospital, Paris, France; 6 Université de Bourgogne, CHU Dijon-Bourgogne, INSERM U1231, Dijon, France

**Keywords:** negative hyperselection, cetuximab, adjuvant, molecular hyperselection, colon cancer

## Abstract

**Background:**

The paradigm of molecular negative hyperselection, beyond *RAS* and *BRAF*, identifies metastatic colorectal cancer (mCRC) patients with the greatest benefit from anti-epidermal growth factor receptor (anti-EGFR) agents. We hypothesize that applying this model to stage III colon cancer (CC) might identify a subgroup deriving benefit from anti-EGFRs in the adjuvant setting.

**Patients and methods:**

We used a ‘simplified PRESSING panel’ grouping genomic alterations related to anti-EGFR primary resistance in mCRC, to select the proficient mismatch repair or microsatellite stable (pMMR/MSS) population of the randomized phase III PETACC-8 clinical trial, testing adjuvant FOLFOX ± cetuximab in resectable stage III CC. Patients without any tumor alterations in *RAS*/*BRAF*, or the simplified PRESSING panel were defined as ‘hyperselected’ (HS), whereas those with at least one such alteration were considered ‘gene altered’ (GA).

**Results:**

Of 1421 eligible patients, 536 (37.7%) were HS and 885 (62.2%) GA. HS patients were more frequently male, with distal tumor of lower stage and grade. HS patients showed improved 5-year relapse-free survival (RFS) (81% versus 68%) and overall survival (OS) (89% versus 77%) as compared with GA patients. A statistically significant interaction was observed for RFS between molecular profile and treatment (*P* = 0.05). Among HS patients, adding cetuximab to FOLFOX yielded a 5-year RFS of 83% versus 78% with FOLFOX alone [hazard ratio (HR) 0.81, 95% confidence interval (CI) 0.55-1.19, *P* = 0.28]. Adding distal tumor location further improved the HR to 0.72 (*P* for interaction = 0.03). No significant interaction for OS was observed. In GA patients, the addition of cetuximab to FOLFOX was detrimental for RFS and OS.

**Conclusions:**

HS was prognostic for RFS and OS in patients with resected stage III pMMR/MSS CC and associated with a trend toward improved RFS with the addition of cetuximab to adjuvant FOLFOX. Conversely, cetuximab seems detrimental in patients with GA tumors. These data may help to improve patient selection in future adjuvant clinical trials testing anti-EGFR therapies.

## Highlights

• Molecular negative hyperselection is prognostic for RFS and OS in resected stage III pMMR/MSS colon cancer (CC).
• The addition of cetuximab to adjuvant FOLFOX improves RFS in patients with hyperselected stage III pMMR/MSS CC.
• RFS with adjuvant cetuximab + FOLFOX is further improved in hyperselected patients with distal primary tumor location.
• Adjuvant cetuximab + FOLFOX is associated with a detrimental effect on RFS and OS in patients with gene altered CC.

## Introduction

A multimodal treatment based on surgery followed by fluoropyrimidines plus oxaliplatin adjuvant chemotherapy is the cornerstone in the search for a cure for patients with stage III colon cancer (CC) and has been the recommended treatment since 2004 in this population.^1^, ^2^, ^3^ However, up to 30% of patients with stage III CC still experience disease recurrence despite this multimodal treatment^4^ and subsequent clinical trials have failed to demonstrate improved outcomes with the addition of biological agents to the adjuvant chemotherapy backbone^5^, ^6^, ^7^, ^8^, ^9^ or by replacing oxaliplatin with irinotecan.^10^, ^11^, ^12^ Specifically, two phase III randomized clinical trials testing the efficacy of the anti-epidermal growth factor receptor (anti-EGFR) agent cetuximab in addition to 5-fluorouracil and oxaliplatin (FOLFOX) in the adjuvant setting for patients with *KRAS* exon 2 wild-type resectable stage III CC have yielded negative results.^7^^,^^8^ However, these trials were conducted many years ago when our understanding of the negative predictive role of genomic alterations, beyond selected *KRAS* mutations, for the efficacy of anti-EGFRs was incomplete.

In the past 10 years the treatment algorithm for patients with metastatic colorectal cancer (mCRC) has shifted greatly toward more personalized treatments based on patient and tumor molecular characteristics.^13^^,^^14^ Patients with left-sided, *RAS/BRAF* wild-type and proficient mismatch repair or microsatellite stable (pMMR/MSS) mCRC are currently considered the optimal candidates to receive a treatment with anti-EGFRs alone or in combination with chemotherapy, given the negative predictive role of *RAS* and *BRAF* mutations, microsatellite instability high (MSI-H) status and right-sided primary tumor location.^15^, ^16^, ^17^, ^18^, ^19^, ^20^ Furthermore, increasing evidence sustains the paradigm of negative hyperselection, including less frequent molecular alterations related to anti-EGFR resistance tested on tumor tissue^21^, ^22^, ^23^ or circulating tumor DNA (ctDNA),^24^^,^^25^ enabling better identification of patients with *RAS* and *BRAF* wild-type mCRC as candidates for anti-EGFRs. In a prospective case-control study involving patients with *RAS* and *BRAF* wild-type mCRC, the tissue-based ‘PRESSING panel’ was developed, to group various rarer molecular alterations related to anti-EGFR primary resistance (HER2 mutations and amplifications, MET amplifications, *ALK/ROS1/NTRK1-3/RET* fusions and *PIK3CA* exon *20/AKT1/PTEN* mutations).^21^ The panel was subsequently validated in a prespecified exploratory analysis^22^ and in a translational study^23^ of two different first-line randomized clinical trials testing the combination of the anti-EGFR panitumumab with FOLFOX or 5-fluorouracil. Also, primary tumor location was consistently identified as a surrogate marker of tumor biology, as these alterations were enriched in tumors primarily located in the proximal colon, and they frequently co-cluster with mismatch repair deficient (dMMR)/MSI-H status.

In the curative setting, a *post hoc* analysis of the PETACC-8 clinical trial testing adjuvant FOLFOX ± cetuximab in stage III CC showed a trend toward better survival outcomes with FOLFOX + cetuximab compared with FOLFOX alone in patients selected for the absence of extended *RAS* (*KRAS* and *NRAS* exon 2, 3, 4) and *BRAF* (exons 11 and 15) mutations.^26^

We hypothesize that extending the paradigm of negative hyperselection in stage III CC patients enrolled in the PETACC-8 clinical trial beyond *RAS/BRAF* by means of a simplified PRESSING panel, in addition to MSI status and primary tumor location selection, may identify a subgroup deriving greater benefit from anti-EGFR agents also in the adjuvant setting.

## Methods

### Patient population

PETACC-8 (NCT00265811) was a phase III, open label, randomized clinical trial enrolling patients with complete (R0) resection of histologically proven stage III colon adenocarcinoma to receive 6 months of either FOLFOX or FOLFOX + cetuximab, as described before.^7^ The trial was conducted from December 2005 to November 2009 and the protocol was amended in June 2008 to enroll only patients with *KRAS* exon 2 wild-type tumors, and the sample size was increased to maintain the power of statistical analyses.

All patients included in this *post hoc* analysis of the PETACC-8 clinical trial had previously provided specific written informed consent for the planned translational program of the trial.

### Molecular analyses and primary tumor location

We restricted our analysis to the biomarker evaluable population of the PETACC-8 clinical trial with pMMR/MSS status to avoid potential confounding due to the prognostic impact of dMMR/MSI-H status in stage III CC.^27^ Furthermore, considering the recent results of immune checkpoint inhibitors in early-stage dMMR/MSI-H CC, the relevance of evaluating adjuvant anti-EGFR therapy in this subgroup remains limited. *KRAS* and *NRAS* exons 2, 3, and 4 mutations, *BRAF* exons 11 and 15 mutations were assessed by polymerase chain reaction or next generation sequencing and MMR/MSI status by immunohistochemistry (IHC) and/or polymerase chain reaction, as previously described.^26^^,^^28^ The simplified PRESSING panel adopted in this analysis was based on the PRESSING panel previously developed in the metastatic setting^21^, ^22^, ^23^ and included all the alterations of the panel that were assessed and available from the PETACC-8 translational program: *PI3KCA* exon 20 mutations, *PTEN* inactivating mutations, *MAP2K1* mutations, *AKT1* mutations, *HER2* mutations and overexpression/amplifications. Gene mutations were assessed with a dedicated panel of 92 amplicons (lon AmpliSeq Colon-Lung Cancer Research Panel version 2; Life Technologies, Carlsbad, CA), covering >500 hotspot mutations in 22 genes. HER2 amplification was assessed by IHC or fluorescent *in situ* hybridization as previously described.^29^ Unfortunately, *MET* amplification and *ALK/ROS1/NTRK/RET* fusions that were part of the original PRESSING panel and assessed by IHC screening were not available in PETACC-8. Even when 3′-RNA sequencing was available as previously described in the PETACC-8 translational cohort,^30^ it did not allow the detection of fusions.

Patients with tumors bearing at least one alteration among *KRAS, NRAS, BRAF* or one of those included in the simplified PRESSING panel were classified as ‘gene altered’ (GA), whereas those with no alterations, when a complete assessment of all the genes of interest was carried out, were classified as ‘hyperselected’ (HS). Patients with incomplete assessment of *RAS*, *BRAF,* or simplified PRESSING panel alterations were included in the GA group if at least one of the prespecified alterations was found in the genes tested or were excluded from the analyses if no alterations were found in the genes examined.

Primary tumor location was categorized as proximal or distal when on the right or the left of the splenic flexure, respectively.

### Statistical analyses

For baseline comparisons, categorical variables were compared with the chi-square test and continuous variables with standard parametric or non-parametric tests, depending on their normality. Continuous variables are reported as mean (standard deviation, SD) and median (interquartile range, IQR) values. Relapse-free survival (RFS) was defined according to the DATECAN definition as the time from date of randomization to local and/or metastatic recurrence, and/or death due to CC, whichever occurred first.^31^ Overall survival (OS) was defined as the time between randomization and death from any cause. RFS and OS curves were estimated using the Kaplan–Meier method and compared with log-rank tests. Hazard ratios (HR) were estimated with the Cox proportional hazard regression model. A two-sided significance level of 5% was applied for all analyses.

Data were imported and handled with R (v 4.2.0) and R Studio (v 2022.02.3).

## Results

### Study population

**Figure 1:**
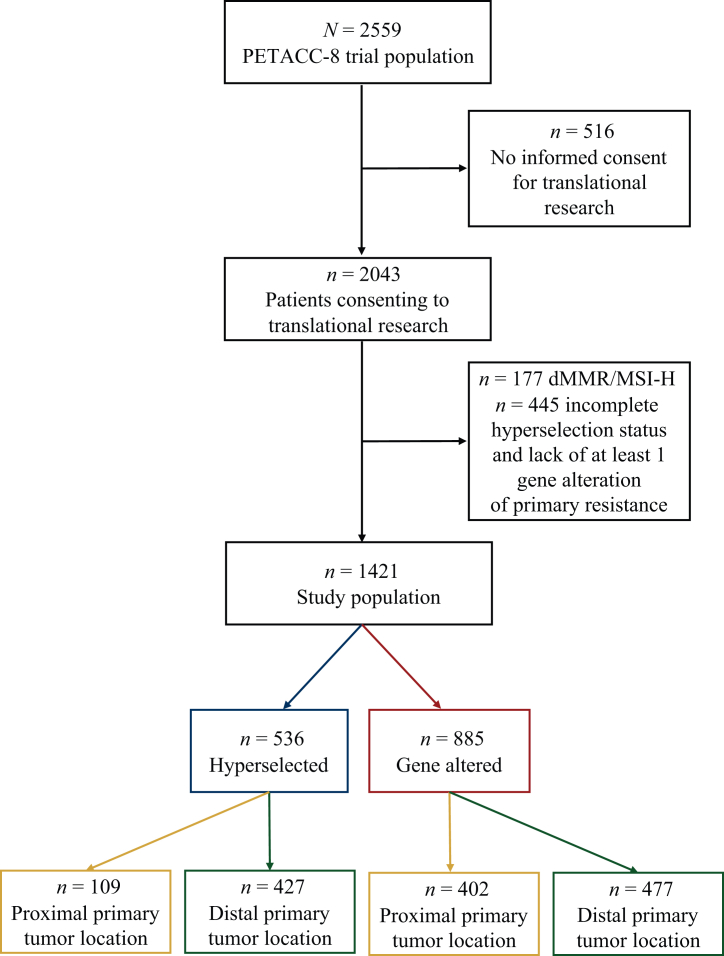
**Study flowchart.** Study flowchart illustrating the size of study population according to molecular profile and primary tumor location.

Among the 2559 patients included in the PETACC-8 clinical trial, 2043 gave their consent to translational research, of whom 177 had dMMR/MSI-H status and 445 had an incomplete HS status with lack of at least one gene alteration of primary resistance, and both were excluded. The study population finally included 1421 patients, of whom 536 (37.7%) and 885 (62.2%) were respectively defined as HS and GA (Figure 1).

To assess comparability between groups, baseline demographic and clinical characteristics of the 1421 patients included in this translational analysis were compared with those of the 622 patients excluded (Supplementary Table S1, available at https://doi.org/10.1016/j.esmoop.2025.105857). We found that only proximal primary tumor location and grade 3-4 were significantly more frequent among excluded patients, consistent with the enrichment of dMMR/MSI-H cases in this population. Moreover, baseline characteristics of the 1421 patients included in this analysis were also similar to those of the full PETACC-8 clinical trial population (Supplementary Table S2, available at https://doi.org/10.1016/j.esmoop.2025.105857).

Patient characteristics are shown in Table 1. As compared with GA, patients with HS tumors were more frequently male (64% versus 54%), with a distal primary tumor location (80% versus 54%), lower T stage (T3-4 87% versus 92%), N stage (N2 34% versus 40%), stage risk (pT4 and/or N2 44% versus 51%) and grade (G3-4 12% versus 19%) according to the American Joint Committee on Cancer (AJCC) TNM 7th Edition. Treatment with FOLFOX + cetuximab was received by a similar proportion of patients in the two groups (49% for HS and 51% for GA).

**Table 1:** Baseline patient characteristics

Characteristic	Molecular profile		*P* value^b^
	Hyperselected	Gene altered	
	*n* = 536	*n* = 885	
**Age, years (**median [IQR]**)**^a^	61 (54-67)	61 (54-67)	>0.9
**Sex, *n* (%)**			**<0.001**
Female	192 (36)	410 (46)	
Male	344 (64)	475 (54)	
**ECOG PS, *n* (%)**			0.2
0	431 (83)	679 (89)	
1-2	90 (17)	173 (20)	
Unknown	15	33	
**Primary tumor location, *n* (%)**			**<0.001**
Proximal	109 (20)	402 (46)	
Distal	427 (80)	477 (54)	
Unknown	0	6	
**Obstruction or perforation, *n* (%)**			0.2
No	442 (82)	705 (80)	
Yes	94 (18)	180 (20)	
**Grade**^c^**, *n* (%)**			**0.002**
1-2	465 (88)	710 (81)	
3-4	66 (12)	164 (19)	
Unknown	5	11	
**pT**^c^**, *n* (%)**			**0.003**
pT1-2	69 (13)	71 (8)	
pT3-4	467 (87)	814 (92)	
**pN**^c^**, *n* (%)**			**0.012**
pN1	356 (66)	529 (60)	
pN2	180 (34)	356 (40)	
**Stage risk**^c^**, *n* (%)**			**0.010**
Low risk (pT1-3 and N1)	301 (56)	435 (49)	
High risk (pT4 and/or N2)	235 (44)	450 (51)	
**Treatment, *n* (%)**			0.4
FOLFOX	274 (51)	433 (49)	
FOLFOX + cetuximab	262 (49)	452 (51)	

*P*-values in bold indicate statistical significance.

ECOG, Eastern Cooperative Oncology Group; IQR, interquartile range; PS, performance status.

a Median (IQR).

b Wilcoxon rank sum test; Pearson’s chi-square test.

c According to the American Joint Comittee on Cancer TNM 7th Edition.

Within the subgroups of patients with HS or GA tumors, and distal or proximal tumors, patient characteristics did not differ according to the type of treatment received (Supplementary Tables S3 and S4, available at https://doi.org/10.1016/j.esmoop.2025.105857).

### Molecular alterations of patients with GA tumors

Among patients with GA tumors and available data for each alteration, 71% (630/885) had mutations in *KRAS*, 7% (60/876) in *NRAS,* and 14% (126/883) in *BRAF*. Regarding the simplified PRESSING panel alterations, 8% (66/877) had mutations in *PI3KCA* exon *20*, 4% (37/862) in *PTEN*, 3% (22/878) in *MAP2K1,* and 1% (11/876) in *AKT1*; 1% (9/876) had activating mutations and 4% (34/885) amplification/overexpression in *HER2* (Supplementary Figure S1, available at https://doi.org/10.1016/j.esmoop.2025.105857).

### Prognostic role of the molecular selection

**Figure 2:**
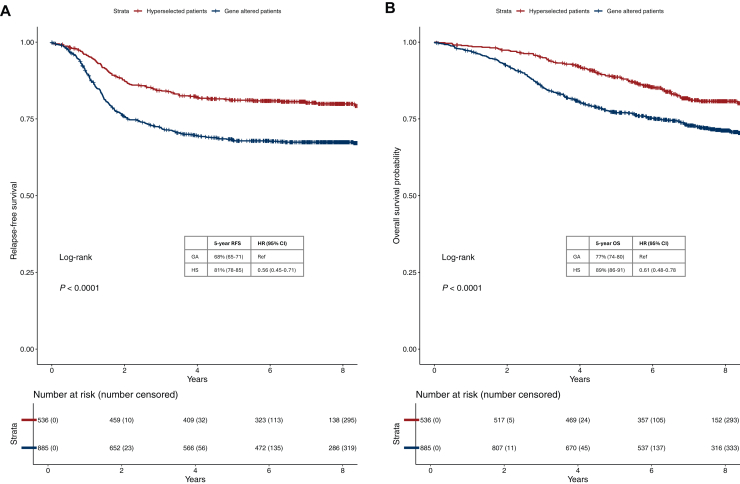
**Kaplan–Meier curves for relapse-free survival (RFS) and overall survival (OS) according to molecular profile.** (A) displays Kaplan–Meier curves for RFS in patients with hyperselected versus gene altered colon cancer; (B) displays Kaplan–Meier curves for OS in patients with hyperselected versus gene altered colon cancer. CI, confidence interval; GA, gene altered; HR, hazard ratio; HS, hyperselected.

We assessed the prognostic value of the molecular selection in the whole cohort. HS was associated with improved RFS as compared with GA (log-rank *P* < 0.001, Figure 2A). Five-year RFS was 81% (95% CI 78% to 85%) in HS patients versus 68% (95% CI 65% to 71%) in GA patients, with an HR of 0.56 (95% CI 0.45-0.71). The prognostic role of HS was confirmed for OS (log-rank *P* < 0.001) with a 5-year OS rate of 89% (95% CI 86% to 91%) versus 77% (95% CI 74% to 80%) and an HR of 0.61 (95% CI 0.48-0.78) (Figure 2B). These results remained statistically significant after adjustment for disease stage risk and grade (HR for RFS 0.60, 95% CI 0.48-0.75, *P* < 0.001 and HR for OS 0.64, 95% CI 0.50-0.81, *P* < 0.001).

### Predictive role of the molecular selection

**Figure 3:**
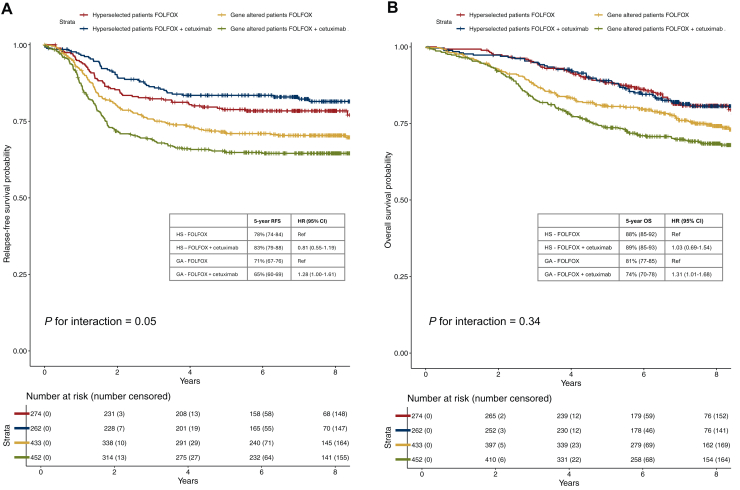
**Kaplan–Meier curves for relapse-free survival (RFS) and overall survival (OS) according to molecular profile and treatment arm. (**A) displays Kaplan–Meier curves for RFS in patients with hyperselected or gene altered colon cancer treated with FOLFOX or FOLFOX + cetuximab; (B) displays Kaplan–Meier curves for OS in patients with hyperselected or gene altered colon cancer treated with FOLFOX or FOLFOX + cetuximab. CI, confidence interval; GA, gene altered; HR, hazard ratio; HS, hyperselected.

A significant interaction with molecular profile and adjuvant treatment arm (with or without cetuximab) was observed for RFS (*P* = 0.05). Patients with HS tumors treated with adjuvant FOLFOX + cetuximab had longer RFS with a 5-year RFS rate of 83% (95% CI 79% to 88%) as compared with 78% (95% CI 74% to 84%) with FOLFOX alone, though not reaching statistical significance (HR 0.81, 95% CI 0.55-1.19, *P* = 0.28). In the GA patient subgroup, a detrimental effect of cetuximab was observed with a 5-year RFS rate of 65% (95% CI 60% to 69%) versus 71% (95% CI 67% to 76%) with FOLFOX alone (HR 1.28, 95% CI 1.00-1.61, *P* = 0.04) (Figure 3A). The interaction between molecular profile and adjuvant treatment arm was not significant for OS (*P* = 0.34) and patients with HS tumors had comparable OS when treated with or without adjuvant cetuximab (5-year OS 89% and 88%, respectively; HR 1.03, 95% CI 0.69-1.54, *P* = 0.87). A detrimental effect of cetuximab was still observed in OS for patients with GA tumors with a 5-year OS rate of 74% (95% CI 70% to 78%) for treatment with FOLFOX + cetuximab as compared with 81% (95% CI 77% to 85%) with FOLFOX alone (HR 1.31, 95% CI 1.01-1.68, *P* = 0.04) (Figure 3B).

**Figure 4:**
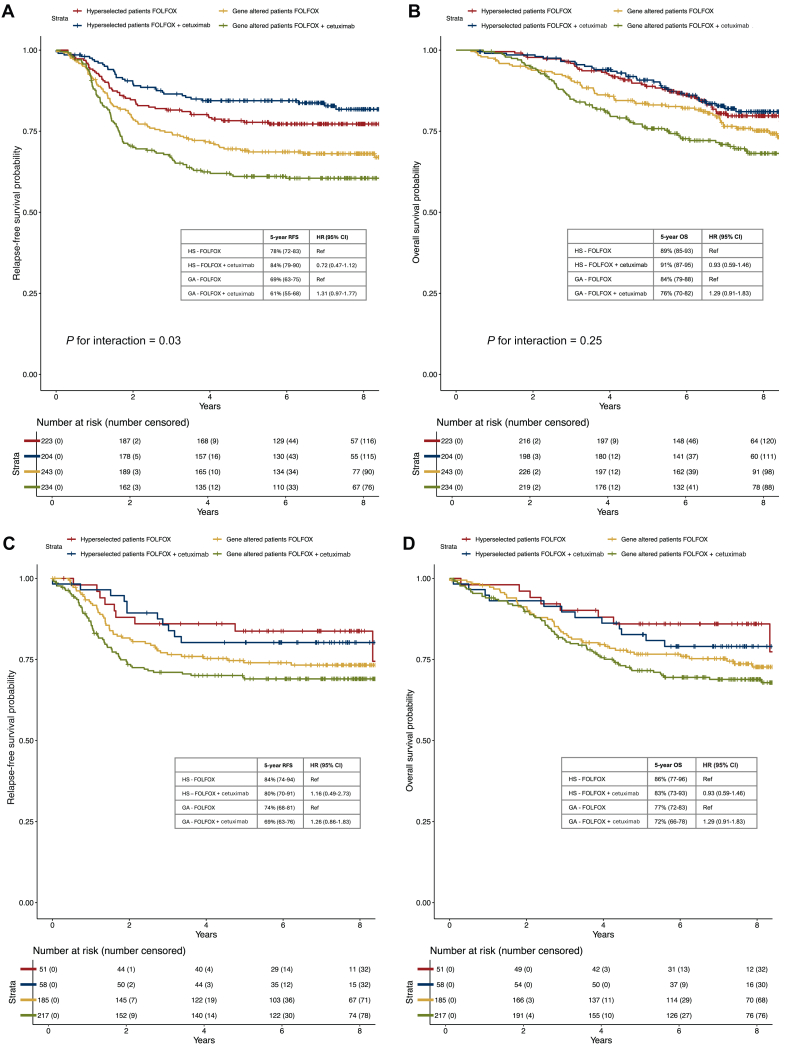
**Kaplan–Meier curves for relapse-free survival (RFS) and overall survival (OS) according to molecular profile and treatment arm in patients with distal or proximal primary tumor location.** (A and B) display Kaplan–Meier curves for RFS and OS in patients with hyperselected or gene altered distal colon cancer treated with FOLFOX or FOLFOX + cetuximab; (C and D) display Kaplan–Meier curves for RFS and OS in patients with hyperselected or gene altered proximal colon cancer treated with FOLFOX or FOLFOX + cetuximab. CI, confidence interval; GA, gene altered; HR, hazard ratio; HS, hyperselected.

Adding primary tumor location to the selection further improved benefit from adjuvant cetuximab for patients with HS tumors. Patients with a distal primary tumor location and HS molecular status achieved a 5-year RFS of 84% (95% CI 79% to 90%) with adjuvant FOLFOX + cetuximab as compared with 78% (95% CI 72% to 83%) with FOLFOX alone (HR 0.72, 95% CI 0.47-1.12, *P* = 0.15; *P* for interaction for RFS = 0.03, Figure 4A). Similarly to the population not selected for primary tumor location, OS did not differ according to adjuvant treatment arm among patients with HS tumors (5-year OS 91% and 89% with or without cetuximab, respectively, HR 0.93, 95% CI 0.59-1.46, *P* = 0.74, Figure 4B) and interaction was not statistically significant (*P* for interaction for OS = 0.25). A trend to a detrimental effect of adjuvant cetuximab was still observed in patients with GA distal primary tumors for RFS (5-year RFS 61% versus 69% with or without cetuximab, respectively; HR 1.31, 95% CI 0.97-1.77, *P* = 0.08, Figure 4A) and for OS (5-year OS 76% versus 84% with or without cetuximab, respectively; HR 1.29, 95% CI 0.91-1.83, *P* = 0.16, Figure 4B).

Conversely, in patients with a proximal primary tumor location and HS tumors, the addition of adjuvant cetuximab to FOLFOX was not beneficial in RFS (80% versus 84% with and without cetuximab, respectively; HR 1.16, 95% CI 0.49-2.73, *P* = 0.74, Figure 4C) or in OS (83% versus 86% with and without cetuximab, respectively; HR 0.93, 95% CI 0.59-1.46, *P* = 0.74, Figure 4D), but was associated with a trend to a detrimental effect in patients with proximal GA tumors both for RFS (5-year RFS 69% versus 74% with and without cetuximab, respectively, HR 1.26, 95% CI 0.86-1.83, *P* = 0.23, Figure 4C) and for OS (5-year OS 72% versus 77% with and without cetuximab, respectively; HR 1.29, 95% CI 0.91-1.83, *P* = 0.16, Figure 4D).

## Discussion

Deeper understanding of the molecular landscape of mCRC has led to the identification of key prognostic and predictive biomarkers that have reshaped therapeutic management through the adoption of targeted strategies that positively impact patient outcomes. In contrast, the standard treatment of stage III CC has remained largely unchanged, despite evidence supporting the prognostic relevance of several biomarkers also in this setting.^27^^,^^29^^,^^32^ As the goal is cure, improving patient outcomes is critical and integrating precision oncology in earlier disease stages of CC guided by insights from the metastatic setting holds promise.

To this aim, it is important to re-evaluate the results of older adjuvant trials investigating anti-EGFR therapies relying only on *KRAS* mutational status. In mCRC, the paradigm of negative hyperselection, combined with left-sided tumor location, has consistently identified patients deriving the greatest benefit from EGFR inhibition.^21^, ^22^, ^23^, ^24^, ^25^ Furthermore, novel EGFR-targeting agents, such as bispecific EGFR-MET targeting antibodies, have given encouraging preliminary results in *RAS* and *BRAF* wild-type mCRC,^33^ and may be used, in the future, to enhance EGFR inhibition in curative-intent strategies, upon appropriate molecular selection.

Based on these premises, in this *post hoc* analysis of the phase III PETACC-8 clinical trial we applied the paradigm of molecular negative hyperselection in the curative setting and assessed its prognostic and predictive value in patients with resected stage III pMMR/MSS CC treated with adjuvant FOLFOX ± cetuximab. We demonstrate that the absence of the molecular alterations included in the simplified PRESSING panel together with *RAS* and *BRAF* wild-type status is associated with improved RFS and OS in stage III pMMR/MSS CC patients undergoing multimodal curative management and identifies a population with a possibly reduced recurrence risk when treated with the addition of cetuximab to standard adjuvant FOLFOX.

A pooled analysis of >7000 patients from seven randomized clinical trials in stage III CC, including PETACC-8, strongly demonstrated that patients with pMMR/MSS CC and absence of *RAS* and *BRAF* mutations have the best RFS (72.9% at 5 years) and OS (73.6% at 8 years).^32^ In our study, using a larger panel of alterations we demonstrated a remarkable 5-year OS of 88% for patients with pMMR/MSS CC and no detectable resistance alterations. Clinical and histopathological characteristics of reduced aggressiveness, such as lower tumor stage and grade at diagnosis, were observed in the baseline characteristics of patients with HS tumors compared with those with GA. The prognostic value of HS was maintained after adjustment for these characteristics in multivariable analysis. Our findings should inform the design of future clinical trials in stage III CC, where the prognostic value of these alterations could guide stratification and statistical hypotheses tailored to these two distinct patient populations.

We observed a significant interaction for RFS between hyperselection status and treatment arm (*P* for interaction = 0.05). The addition of cetuximab to adjuvant FOLFOX resulted in an absolute 5% gain in RFS at 5 years in patients with HS tumors (83% versus 78%), a magnitude comparable to the clinically relevant benefit obtained from the addition of oxaliplatin to 5-fluorouracil in stage III CC.^4^ Adding primary tumor location to negative hyperselection improved the absolute potential benefit from cetuximab, moving from an HR of 0.81 for RFS in the overall population to 0.72 for patients with HS distal primary tumors (*P* for interaction = 0.03).

This improvement, however, did not reach statistical significance in the overall population (*P* = 0.28) or in patients with distal primary tumors (*P* = 0.15), likely due to the *post hoc* nature of the analysis and limited statistical power in small number subgroups. Additionally, no OS difference was observed (89% and 88% at 5 years with and without cetuximab, respectively), which may reflect the need for a larger sample size to detect survival differences in a population with already high cure rates. Moreover, treatments after progression are not known for this population and may have significantly impacted OS.

Primary tumor location proves to be a surrogate of tumor biology given the enrichment of right sidedness among patients with a GA tumor profile, as already described in the metastatic setting.^21^, ^22^, ^23^, ^24^^,^^34^^,^^35^ Notably, in our study, patients with proximal tumors derived no benefit from cetuximab, even when negatively hyperselected. In contrast, the PARADIGM trial found a trend toward greater benefit from panitumumab over bevacizumab in the metastatic setting in HS patients with right primary tumor location.^24^ This might suggest that the simplified panel used here may not fully capture resistance mechanisms in right-sided locally advanced tumors, warranting exploration of additional genomic and epigenomic markers.

Neoadjuvant chemotherapy is emerging as a promising strategy to improve outcomes of patients with CC in the curative setting.^36^ In the extended biomarker analysis of the panitumumab arm of the FOxTROT trial of neoadjuvant chemotherapy in locally advanced CC, a high expression of the EGFR ligands AREG and EREG was predictive of improved disease free survival and OS in patients with *RAS/BRAF* wild-type tumors, albeit in a small patient cohort.^37^ Unfortunately, AREG/EREG expression was not assessed in PETACC-8. Nevertheless, AREG/EREG expression may offer further refinement in selecting candidates for anti-EGFR therapy both in distal and proximal tumor locations and should be evaluated together with genomic negative hyperselection. The ongoing ARIEL trial (ISRCTN11061442) is expected to provide prospective validation of high AREG/EREG expression in right-sided, *RAS* wild-type mCRC,^38^ potentially also providing useful information for future (neo)adjuvant trials using new-generation anti-EGFR agents.

Conversely, for patients with GA tumors, cetuximab was detrimental for both RFS and OS, underscoring the need to exclude these patients from further anti-EGFR development in the curative setting. Interestingly, most of these molecular alterations are potentially actionable (e.g. *KRAS*, *BRAF*, HER2, *PI3KCA*) and the development of matched targeted strategies should be favored.

Considering the *post hoc* nature of our subgroup analyses and a significant attrition in patient numbers, these findings remain hypothesis-generating. We have used a simplified PRESSING panel based on the available tumor molecular alterations for the cohort of patients enrolled in the PETACC-8 clinical trial, but the optimal patient selection for EGFR blocking strategies in the adjuvant setting still needs to be defined. Our simplified panel includes the most frequent secondary alterations of primary resistance to anti-EGFRs and might be more cost effective in clinical practice. Indeed, this approach raises the question of the feasibility of extended genomic profiling at diagnosis. With the increasing number of neoadjuvant platform trials of targeted treatments matched with tumor molecular characteristics (NCT05845450, NCT05706779, ISRCTN83842641), and the adoption of tumor-informed ctDNA assays, the paradigm of molecular characterization in CC will probably soon change. Due to sample size limitations and the rarity of the alterations of the simplified PRESSING panel compared with the more common *RAS* and *BRAF* mutations in CC, we could not separately assess the contribution of the panel itself to anti-EGFR predictiveness in this population. Future studies should involve a larger population already selected for *RAS* and *BRAF* wild-type and pMMR/MSS status to validate its role.

In conclusion, our results show that molecular hyperselection makes it possible to identify groups of patients with different prognoses, for whom anti-EGFRs could be beneficial or, on the contrary, detrimental. With the rapid development of tumor-informed ctDNA assays that will provide a complete molecular profile of the disease from the earliest stages, this simplified PRESSING panel should, in conjunction with primary tumor location and potentially other epigenetic markers, prove useful for improving patient selection in future adjuvant trials evaluating new-generation anti-EGFRs.
