# Supplementary material for: Negative hyperselection of patients with stage III colon cancer receiving anti-EGFR-based adjuvant treatment
Source: ESMO Open. 2025 Oct 29;10(11):105857. doi: 10.1016/j.esmoop.2025.105857 (PMC12607000; doi:10.1016/j.esmoop.2025.105857)
Supplement: Supplementary material [file mmc1.docx]

**Supplementary material**

**Negative hyperselection of patients with stage III colon cancer receiving anti-EGFR─based adjuvant treatment.**

Margherita Ambrosini, Héléne Blons, Simon Garinet, Claire Mulot, Delphine Le Corre, Sophie Mouillet-Richard, Filippo Pietrantonio, Marine Sroussi, Come Lepage, Pierre Laurent-Puig, Julien Taieb.

**Supplementary Table 1. Baseline characteristics of patients included in the study population and of patients excluded due to dMMR/MSI-H status or incomplete hyperselection status and lack of at least one gene alteration of primary resistance.**

**Supplementary Table 2. Baseline characteristics of patients included in the study population and of patients enrolled in PETACC-8 clinical trial.**

**Supplementary Table 3. Baseline characteristics of patients with hyperselected or gene altered molecular profile according to type of treatment.**

**Supplementary Table 4. Baseline characteristics of patients with distal or proximal primary tumor according to type of treatment.**

**Supplementary Figure 1. Heatmap of molecular alterations of patients with gene altered tumors.**

**Supplementary Table 1. Baseline characteristics of patients included in the study population and of patients excluded due to dMMR/MSI-H status or incomplete hyperselection status and lack of at least one gene alteration of primary resistance.**

|  | **Patients consenting to the translational research**  N=2043 | | |
| --- | --- | --- | --- |
| **Characteristic** | **Study population**  N = 1421^1^ | **Patients excluded**  N=622^1^ | ***p-value****^2^* |
| **Age [median (IQR)]** | 61 (54, 67) | 60 (52, 67) | *0.078* |
| **Sex** |  |  | *0.5* |
| Female | 601 (42%) | 273 (44%) |  |
| Male | 820 (58%) | 349 (56%) |  |
| **ECOG PS** |  |  | *0.085* |
| 0 | 1109 (81%) | 502 (84%) |  |
| 1-2 | 263 (19%) | 95 (16%) |  |
| Unknown | 49 | 25 |  |
| **Primary tumor location** |  |  | ***<0.001*** |
| Proximal | 509 (36%) | 290 (47%) |  |
| Distal | 906 (64%) | 329 (53%) |  |
| Unknown | 6 | 3 |  |
| **Obstruction or perforation** |  |  | *0.8* |
| No | 1147 (81%) | 505 (81%) |  |
| Yes | 274 (19%) | 117 (19%) |  |
| **Grade** |  |  | ***<0.001*** |
| G1-2 | 1176 (84%) | 468 (76%) |  |
| G3-4 | 229 (16%) | 145 (24%) |  |
| Unknown | 16 | 9 |  |
| **pT** |  |  | *>0.9* |
| pT1 | 41 (3%) | 19 (3%) |  |
| pT2 | 99 (7%) | 46 (7%) |  |
| pT3 | 990 (70%) | 423 (68%) |  |
| pT4 | 291 (20%) | 131 (21%) |  |
| Unknown | 0 | 3 |  |
| **pN** |  |  | *>0.9* |
| pN1 | 886 (62%) | 389 (63%) |  |
| pN2 | 535 (38%) | 233 (37%) |  |
| **Stage risk** |  |  | *0.7* |
| Low risk  (pT1-3 and N1) | 737 (52%) | 315 (51%) |  |
| High risk  (pT4 and/or N2) | 684 (48%) | 305 (49%) |  |
| Unknown | 0 | 2 |  |
| **Treatment arm** |  |  | *0.9* |
| FOLFOX | 706 (50%) | 311 (50%) |  |
| FOLFOX + Cetuximab | 715 (50%) | 311 (50%) |  |

**Supplementary Table 2. Baseline characteristics of patients included in the study population and of patients enrolled in PETACC-8 clinical trial.**

| **Characteristic** | **Study population**  N = 1421^1^ | **PETACC-8**  **population**  N=2559^1^ |  |
| --- | --- | --- | --- |
| **Age [median (IQR)]** | 61 (54, 67) | 60 (53, 67) |  |
| **Sex** |  |  |  |
| Female | 601 (42%) | 1097 (43%) |  |
| Male | 820 (58%) | 1462 (57%) |  |
| **ECOG PS** |  |  |  |
| 0 | 1109 (81%) | 1611 (82%) |  |
| 1-2 | 263 (19%) | 358 (18%) |  |
| Unknown | 49 | 590 |  |
| **Primary tumor location** |  |  |  |
| Proximal | 509 (36%) | 968 (38%) |  |
| Distal | 906 (64%) | 1552 (62%) |  |
| Unknown | 6 | 39 |  |
| **Obstruction or perforation** |  |  |  |
| No | 1147 (81%) | 2063 (81%) |  |
| Yes | 274 (19%) | 496 (19%) |  |
| **Grade** |  |  |  |
| G1-2 | 1176 (84%) | 2054 (81%) |  |
| G3-4 | 229 (16%) | 472 (19%) |  |
| Unknown | 16 | 33 |  |
| **pT** |  |  |  |
| pT1 | 41 (3%) | 71 (3%) |  |
| pT2 | 99 (7%) | 194 (8%) |  |
| pT3 | 990 (70%) | 1768 (69%) |  |
| pT4 | 291 (20%) | 522 (20%) |  |
| Unknown | 0 | 4 |  |
| **pN** |  |  |  |
| pN1 | 886 (62%) | 1597 (62%) |  |
| pN2 | 535 (38%) | 962 (38%) |  |
| **Stage risk** |  |  |  |
| Low risk  (pT1-3 and N1) | 737 (52%) | 1316 (52%) |  |
| High risk  (pT4 and/or N2) | 684 (48%) | 1241 (48%) |  |
| **Treatment arm** |  |  |  |
| FOLFOX | 706 (50%) | 1279 (50%) |  |
| FOLFOX + Cetuximab | 715 (50%) | 1280 (50%) |  |
| ^1^Median (IQR); n (%) | | |  |
| ^2^Wilcoxon rank sum test; Pearson's Chi-squared test | | |  |

**Supplementary Table 3. Baseline characteristics of patients with hyperselected or gene altered molecular profile according to type of treatment.**

|  | **Hyperselected**  N = 536 | | | | **Gene altered**  N = 885 | | | |
| --- | --- | --- | --- | --- | --- | --- | --- | --- |
| **Characteristic** | **Treatment** | | | | | | |  |
|  | **FOLFOX**  N = 274^1^ | **FOLFOX +**  **cetuximab**  N = 262^1^ | ***p-value****^2^* | **FOLFOX**  N = 433^1^ | | **FOLFOX + cetuximab**  N = 452^1^ | ***p-value****^2^* |  |
| **Age [median (IQR)]** | 60 (54, 67) | 61 (54, 68) | *0.3* | 61 (55, 67) | | 60 (53, 66) | ***0.038*** |  |
| **Sex** |  |  | *0.2* |  | |  | *0.6* |  |
| Female | 106 (39%) | 86 (33%) |  | 205 (47%) | | 205 (45%) |  |  |
| Male | 168 (61%) | 176 (67%) |  | 228 (53%) | | 247 (55%) |  |  |
| **ECOG PS** |  |  | *>0.9* |  | |  | *0.3* |  |
| 0 | 221 (83%) | 210 (83%) |  | 342 (81%) | | 337 (78%) |  |  |
| 1-2 | 46 (17%) | 44 (17%) |  | 79 (19%) | | 94 (22%) |  |  |
| Unknown | 7 | 8 |  | 12 | | 21 |  |  |
| **Primary tumor location** |  |  | *0.3* |  | |  | *0.15* |  |
| Proximal | 51 (19%) | 58 (22%) |  | 185 (43%) | | 217 (48%) |  |  |
| Distal | 223 (81%) | 204 (78%) |  | 243 (57%) | | 234 (52%) |  |  |
| Unknown | 0 | 0 |  | 5 | | 1 |  |  |
| **Obstruction or perforation** |  |  | *0.6* |  | |  | *0.5* |  |
| No | 228 (89%) | 214 (86%) |  | 349 (80%) | | 356 (79%) |  |  |
| Yes | 46 (11%) | 48 (14%) |  | 84 (20%) | | 96 (21%) |  |  |
| **Grade** |  |  | *0.5* |  | |  | *0.4* |  |
| G1-2 | 240 (83%) | 225 (87%) |  | 341 (80%) | | 369 (82%) |  |  |
| G3-4 | 31 (17%) | 35 (13%) |  | 85 (20%) | | 79 (18%) |  |  |
| Unknown | 3 | 2 |  | 7 | | 4 |  |  |
| **pT** |  |  | *0.4* |  | |  | *0.12* |  |
| pT1-2 | 32 (12%) | 37 (14%) |  | 41 (9%) | | 30 (7%) |  |  |
| pT3-4 | 242 (88%) | 225 (86%) |  | 392 (91%) | | 422 (93%) |  |  |
| **pN** |  |  | *>0.9* |  | |  | *0.5* |  |
| pN1 | 182 (66%) | 174 (66%) |  | 264 (61%) | | 265 (59%) |  |  |
| pN2 | 92 (34%) | 88 (34%) |  | 169 (39%) | | 187 (41%) |  |  |
| **Stage risk** |  |  | *0.4* |  | |  | *0.8* |  |
| Low risk  (pT1-3 and N1) | 159 (58%) | 142 (54%) |  | 215 (50%) | | 220 (49%) |  |  |
| High risk  (pT4 and/or N2) | 115 (42%) | 120 (46%) |  | 218 (50%) | | 232 (51%) |  |  |
| *^1^Median (IQR); n (%)* | | | |  | | | |  |
| *^2^Wilcoxon rank sum test; Pearson's Chi-squared test* | | | |  |  |  |  |  |

**Supplementary Table 4. Baseline characteristics of patients with distal or proximal primary tumor according to type of treatment.**

|  | **Distal primary tumor**  N = 904 | | | | **Proximal primary tumor**  N = 511 | | |
| --- | --- | --- | --- | --- | --- | --- | --- |
| **Characteristic** | **Treatment** | | | | | | |
|  | **FOLFOX**  N = 466^1^ | **FOLFOX +**  **cetuximab**  N = 438^1^ | ***p-value****^2^* | **FOLFOX**  N = 236^1^ | | **FOLFOX +**  **cetuximab**  N = 275^1^ | ***p-value****^2^* |
| **Age [median (IQR)]** | 60 (54, 66) | 60 (52, 66) | *0.3* | 62 (56, 67) | | 61 (55, 67) | *0.5* |
| **Sex** |  |  | *0.4* |  | |  | *0.3* |
| Female | 209 (45%) | 184 (42%) |  | 102 (43%) | | 107 (39%) |  |
| Male | 257 (55%) | 254 (58%) |  | 134 (57%) | | 168 (61%) |  |
| **ECOG PS** |  |  | *0.8* |  | |  | *0.2* |
| 0 | 368 (81%) | 338 (80%) |  | 191 (84%) | | 209 (79%) |  |
| 1-2 | 88 (19%) | 84 (20%) |  | 36 (16%) | | 54 (21%) |  |
| Unknown | 10 | 16 |  | 9 | | 12 |  |
| **Grade** |  |  | *>0.9* |  | |  | *0.4* |
| G1-2 | 401 (87%) | 378 (87%) |  | 176 (76%) | | 215 (79%) |  |
| G3-4 | 59 (13%) | 57 (13%) |  | 56 (24%) | | 57 (21%) |  |
| Unknown | 6 | 3 |  | 4 | | 3 |  |
| **Obstruction or perforation** |  |  | *0.4* |  | |  | *0.6* |
| No | 371 (80) | 338 (77%) |  | 202 (86%) | | 231 (84%) |  |
| Yes | 95 (20%) | 100 (23%) |  | 34 (14%) | | 44 (16%) |  |
| **pT** |  |  | *0.5* |  | |  | *>0.9* |
| pT1-2 | 53 (11%) | 44 (10%) |  | 20 (8.5%) | | 23 (8.4%) |  |
| pT3-4 | 413 (89%) | 394 (90%) |  | 216 (92%) | | 252 (92%) |  |
| **pN** |  |  | *0.8* |  | |  | *0.3* |
| pN1 | 298 (64%) | 283 (65%) |  | 144 (61%) | | 155 (56%) |  |
| pN2 | 168 (36%) | 155 (35%) |  | 92 (39%) | | 120 (44%) |  |
| **Stage risk** |  |  | *0.9* |  | |  | *0.3* |
| Low risk  (pT1-3 and N1) | 246 (53%) | 229 (52%) |  | 125 (53%) | | 132 (48%) |  |
| High risk  (pT4 and/or N2) | 220 (47%) | 209 (48%) |  | 111 (47%) | | 143 (52%) |  |
| **Molecular profile** |  |  | *0.7* |  | |  | *0.6* |
| Gene altered | 243 (52%) | 234 (53%) |  | 185 (78%) | | 217 (79%) |  |
| Hyperselected | 223 (48%) | 204 (47%) |  | 51 (22%) | | 58 (21%) |  |
| *^1^Median (IQR); n (%)*  *^2^Wilcoxon rank sum test; Pearson's Chi-squared test; Fisher's exact test* | | | | | | | |

**Supplementary Figure 1. Heatmap of molecular alterations of patients with gene altered tumors.**
